# Unconventional Use of Intense Pulsed Light

**DOI:** 10.1155/2014/618206

**Published:** 2014-09-03

**Authors:** D. Piccolo, D. Di Marcantonio, G. Crisman, G. Cannarozzo, M. Sannino, A. Chiricozzi, S. Chimenti

**Affiliations:** ^1^Department of Dermatology, University of L'Aquila, Via Vetoio, Coppito 2, 67100 L'Aquila, Italy; ^2^Italian Society of Laser Dermatology (SILD), Via Nicolò dall'Arca 7, 70121 Bari, Italy; ^3^Department of Dermatology, University of Rome, Tor Vergata, Italy; ^4^Department of Dermatology, University of Bologna, Italy; ^5^Laboratory for Investigative Dermatology, The Rockefeller University, New York City, USA

## Abstract

According to the literature, intense pulsed light (IPL) represents a versatile tool in the treatment of some dermatological conditions (i.e., pigmentation disorders, hair removal, and acne), due to its wide range of wavelengths. The authors herein report on 58 unconventional but effective uses of IPL in several cutaneous diseases, such as rosacea (10 cases), port-wine stain (PWS) (10 cases), disseminated porokeratosis (10 cases), pilonidal cyst (3 cases), seborrheic keratosis (10 cases), hypertrophic scar (5 cases) and keloid scar (5 cases), Becker's nevus (2 cases), hidradenitis suppurativa (2 cases), and sarcoidosis (1 case). Our results should suggest that IPL could represent a valid therapeutic support and option by providing excellent outcomes and low side effects, even though it should be underlined that the use and the effectiveness of IPL are strongly related to the operator's experience (acquired by attempting at least one specific course on the use of IPL and one-year experience in a specialized centre). Moreover, the daily use of these devices will surely increase clinical experience and provide new information, thus enhancing long-term results and improving IPL effectiveness.

## 1. Introduction

First introduced in 1990s, intense pulsed light (IPL) was obtained by U.S. Food and Drug Administration (FDA) authorization in 1995 for the treatment of lower-limb telangiectasias.

This polychromatic, noncoherent, and broad-spectrum pulsed light source (xenon lamp) is able to emit light of a wavelength between 390 nm and 1200 nm [1]. Its basic principle consists in the absorption of photons by exogenous or endogenous chromophores within the skin; this transfer of energy to the target structures generates heat and subsequent destruction of the target through a process called selective photothermolysis. The wavelength should be selected in dependence of the absorption peak of the target chromophore and the pulse duration should last less than the thermal relaxation time. This limits the diffusion of heat and damage to surrounding structures.

The main chromophores of the skin, such as haemoglobin, melanin, and water, have a broad absorption spectrum. Through the use of a filter, available from 500 nm to 755 nm, it is possible to select the wavelengths suitable for the established treatment. The IPL's pulse duration may be set within a relatively wide range between 1 and 100 milliseconds, depending on the selected device. In addition, a wide range of treatment parameters, including pulse sequence and pulse delay time, can be customized, thus giving users greater versatility and accuracy [1].

Versatility represents a significant advantage for experienced dermatologists, but it could be a serious limit for nonexperienced physicians and for nonmedical staff since an erroneous selection of the setting can cause serious side effects.

In daily practice, the application of a gel is necessary, as well as direct contact between the handpiece and the skin, although this hinders the local immediate response.

The combination of wavelength, pulse duration, delay, and fluence allows the use of IPL devices in the treatment of several dermatological conditions, such as acne vulgaris, pigmentation disorders, vascular lesions, hirsutism, photodamaged skin, scars and birthmarks, and melasma [2–4].

The authors herein suggest many unconventional uses of IPL in the treatment of different dermatological conditions, such as rosacea (10 cases), port-wine stain (PWS) (10 cases), disseminated porokeratosis (10 cases), pilonidal cyst (3 cases), seborrheic keratosis (10 cases), hypertrophic scar (5 cases) and keloid scar (5 cases), Becker's nevus (2 cases), hidradenitis suppurativa (2 cases), and sarcoidosis (1 case).

*Acne rosacea *or* rosacea* is a chronic dermatitis of unknown aetiology, characterized by erythema, telangiectasias, papules and pustules [5, 6].

*Port-wine stain* is a common congenital vascular malformation occurring in up to 25% of infants [7–10].

*Disseminated porokeratosis* is a localized alteration of keratinization. Clinically, one or more atrophic mainly asymptomatic and sometimes mildly itching plaques surrounded by an hyperkeratotic border (histologically defined as a cornoid lamella) are observed due to a rapid proliferation of atypical keratinocytes [11–14].

*Pilonidal cyst*, also known as* pilonidal sinus* or* sacrococcygeal cyst* (due to its frequent onset in this area), is a cyst containing hair and skin debris [15–19].

*Seborrheic keratosis* is a benign skin lesion of the epidermis, mainly localized on seborrheic areas, in particular, the face and trunk. The most common clinical presentation is a lesion with warty or squamous crusted surface of variable size, coloured yellow-brown or dark-brown with blackish specks, with soft consistency [20, 21].

*Hypertrophic scars and keloids* are a serious physical and psychological dermatological condition for patients. Despite the several studies performed on metabolisms and treatment of wounds and scars, the exact pathogenesis of keloids and hypertrophic scars remains unknown and this makes therapies even more complicated [22].

*Becker's nevus* is a mostly male-predominant birthmark hyperpigmentation, presenting with a unilateral (rarely bilateral), benign hypermelanotic patch usually sited on the shoulder, chest, or lower back. Grouped brown spots with a bizarre border are the common presentation, with hypertrichosis in half of the cases [23, 24].

*Hidradenitis suppurativa* is a common disease, also known as acne inversa, which leads to a chronic relapsing suppurative inflammation of regions where apocrine glands occur, that is, axilla, inguinal folds, perineum, genitalia, and periareolar region. Several predisposing, triggering, and etiologic factors have been encountered (androgenic dysfunction, obesity, etc.); thus, authors agree that aetiology is still unclear. Commonly, the follicles into which the apocrine glands open are plugged by keratin and infections, mainly caused by anaerobic organisms which develop the following stasis and cause cysts that are extremely painful to palpation [25–27].

*Sarcoidosis* is both a systemic and a dermatologic syndrome of unknown etiology which can affect the skin as well the lymph nodes and viscera. The lesions can be single or multiple and can range from macules to large plaques and nodules. Cutaneous involvement is referred to in up to 25% of patients with systemic sarcoidosis. Plaques, maculopapular eruptions, subcutaneous nodules, and lupus pernio can be observed as well as cutaneous manifestations [28–33].

## 2. Material and Methods

58 consecutive patients (32 males and 26 females, mean age 42.7—range 8–83) presenting with nine different dermatological disorders were treated with IPL as an unconventional approach (Table 1). The aim of the study was to verify the efficacy of IPL by comparing the obtained results with results achieved through conventional treatment options (according to the literature) using either clinical or dermoscopic pictures before and after each session. Notably, dermoscopy conducted before treatment confirmed its usefulness in confirming diagnosis and in highlighting specific characteristics of each condition, such as number and calibre of blood vessels, distribution of pigment, and presence of crusts or hairs; thus, it also represents a valid method for outcome assessments [34]. An IPL device (Deka M.E.L.A. Srl, Calenzano, Florence, Italy) with two different handpieces for 500 nm and 550 nm filters was used and set according to the skin type and clinical characteristics of each patient. Dermoscopic images were made in all cases before, immediately after and at distance from each treatment using a special lens for dermoscopy (DermLite Foto, 3GEN LLC, San Juan Capistrano, CA, USA) connected to a digital camera (Canon PowerShot A360).

A soothing cream, a gentle cleansing, and a photoprotection (SPF50) solution were prescribed to each patient after each session.

In the following, the authors describe the IPL scheme treatments and the results obtained for each dermatological condition. Each patient has been informed that at least two sessions up to six sessions, with intervals of approximately 20–30 days, are needed to gain significant results.

*Rosacea*. Ten patients (5 females and 5 males) aged between 38 and 62 years (average age 51.6 years) with Fitzpatrick phototype II-III presented with rosacea, 6 with an erythematotelangiectatic form, 3 with papules and pustules, and only one with rhinophyma.

The telangiectatic component was treated with the 500 nm handpiece, while the papulopustular component was subsequently treated with the 550 nm handpiece (Table 2).

*Port-Wine Stain*. Ten patients (7 males and 3 females) aged between 8 and 52 years (average age 22.1 years) with Fitzpatrick phototype II-III were treated for the presence of a PWS. Lesions were sited on the malar part of the face (3 cases), on the nose (2 cases), on the glabella (1 case), on the upper lip (1 case), on the forehead (1 case), on the posterior part of the neck (1 case), and on the posterior upper-right limb (1 case), respectively. Table 2 shows the IPL setting used in these cases.

*Disseminated Porokeratosis*. Ten patients (8 females and 2 males) aged between 41 and 70 years (average age 58.3 years) with Fitzpatrick phototypes II–IV were treated for the presence of multiple disseminated, atrophic, and slightly itchy plaques with a hyperkeratotic border. The lesions were mainly located on the lower extremities (50%), on the upper extremities (40%), and on the back (10%). Protocol shown on Table 2 has been successfully applied to these patients.

*Pilonidal Cyst*. Three patients (3 males) 18, 22, and 34 years old (average age 24.6 years) presented with a recurrent, inflamed, sore, and swollen cyst localized in the sacrococcygeal region. The lesion of the oldest patient had already been surgically treated. IPL action on hair follicles is well known and we thus suggested the use of this device with the aim of destroying hairs encapsulated within the cyst and hairs in the surrounding area. The anti-inflammatory properties of IPL proved to be effective in reducing the risk of recurrence. We decided to treat the lesion according to the protocol shown on Table 2.

*Seborrheic Keratosis*. Ten patients (6 males and 4 females) aged between 35 and 83 years (average age 61.7 years) with Fitzpatrick phototypes I–III were treated for the presence of multiple disseminated small seborrheic keratoses sited on the face (30%), on the chest (25%), and on the back (45%).

All lesions were treated at intervals of 15–20 days for a total of 4 sessions per case according to the protocol shown in Table 2.

Dermoscopic images were obtained for each case before (also for diagnostic purpose), immediately after, and at a distance from each treatment using the same equipment described above.

*Hypertrophic Scars and Keloids*. Ten patients, 5 presenting with hypertrophic scars (3 males and 2 females aged between 21 and 37 years, average age 30.2 years) and 5 presenting with keloids (3 females and 2 males aged between 27 and 43 years, average age 34.8 years), were treated with both 500 nm (vascular component) and 550 nm (pigmented component) wavelength handpieces. The first sessions with the 550 nm handpiece were carried out for the pigmented component where present. Whereupon, successive treatments with the 500 nm handpiece have been made for treating the vascular component (Table 2).

At least 30 days of rest are required before the subsequent session and a few months are needed to obtain very positive results.

*Becker's Nevus*. A 32-year-old man presented with Becker's nevus sited on his left shoulder blade. Clinically, a hypertrichotic brown patch with irregular edges of 12 cm × 9 cm in size was observed. Successively, a 26-year-old man presented with Becker's nevus without hypertrichosis of 8,5 cm × 8 cm in size and sited on his upper-right chest. In the first case, we decided to first use a 550nm wavelength handpiece with the aim of removing the hair components (Table 2).

After four sessions of IPL at intervals of 40 days, we performed two additional sessions with the aim of treating the hyperpigmented component (Table 2). Only the protocol shown in Table 2 was applied to the second patient since the hypertrichotic component was not present.

*Hidradenitis Suppurativa*. One 38-year-old man, previously treated in a surgical way (clinical stage II (Hurley's staging system) and sartorius score of 36), and one 26-year-old woman presented with hidradenitis suppurativa of the axillary region, bilateral (clinical stage I (Hurley's staging system) and sartorius score of 24).

After four sessions of IPL at intervals of 15–20 days, we performed two additional sessions with 2 pulses of 5 ms and 10 ms separated by a delay of 10 ms and a fluence of 9 J/cm^2^ with the aim of treating the inflammatory component (Table 2).

*Sarcoidosis*. A 26-year-old female presented with three painful, firm, and vascularized nodules sited on the anterior and posterior parts of the pinna and on the helix. Through histopathological examination, a diagnosis of sarcoidosis was posed. The patient had already undergone intralesional corticosteroid therapy without results. Thus, we suggested using the IPL device with the aim of hitting the very prominent (especially on dermoscopic evaluation) vascular component within the lesions.

## 3. Results

In this study, we obtained good outcomes for all the treated patients, who were affected by different dermatological conditions. Our results are summarized as follows.

**Figure 1:**
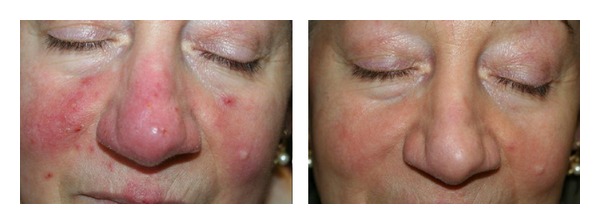
Rosacea: significant results with a significant reduction in vessel number and size and a complete disappearance of papules have been achieved after 4 IPL sessions.

*Rosacea*. Patients required from 2 to 5 sessions, at intervals of approximately 20–30 days, to gain significant results, even though a moderate reduction in vessel number and size and a partial disappearance of papules were observed subsequent to the second session (Figure 1). A 12-month follow-up revealed the complete absence of recurrences and the persistence of the achieved outcomes in 7 of 10 patients (70%) whereas the other 3 patients required a new treatment within the year for the slight relapse of the papulopustular component.

*Port-Wine Stain*. The results were already visible after the end of the first session. Dermoscopy performed before treatment highlighted the number, calibre, and depth of the target vessels. Superficial vessels were hit with greater accuracy by IPL and dermoscopic examinations revealed a change in vessel colour from red to blue immediately after treatment. In cases of high numbers of vessels, erosions and crusts can follow treatment sessions for several days. The number of the treatments required to gain significant results depended on the depth and site of the PWS.

**Figure 2:**
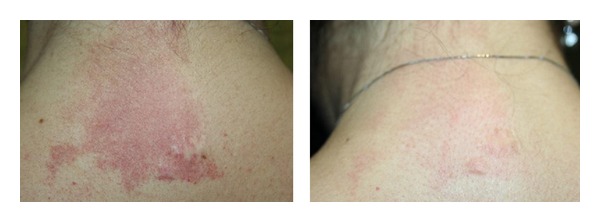
Port-wine stain: after 4 IPL sessions, the patient gained excellent results.

Three out of 10 patients (30%) obtained excellent results (disappearance of PWS), 6 of 10 (60%) obtained good results (disappearance of almost 70% of treated vessels), and only one (10%) obtained a moderate result (disappearance of about 30% of the lesion) (Figure 2). The obtained results, confirmed by dermoscopy, were stable after a follow-up period ranging from 1 to 3 years.

**Figure 3:**
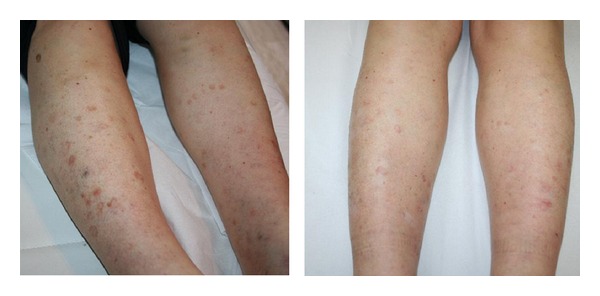
Disseminated porokeratosis: after 4 treatments, an important reduction of the hyperkeratotic edge and a reduction in the intensity of melanin have been observed.

*Disseminated Porokeratosis*. All treated patients showed interesting results, despite the fact that the histology confirmed the persistence of cornoid lamella. In fact, one patient who had shown significant improvements after four sessions presented at the follow-up visit with an important reduction of the hyperkeratotic edge and a reduction in the intensity of melanin (Figure 3); a punch biopsy was performed and the histopathologic examination revealed the persistence of a cornoid lamella.

**Figure 4:**
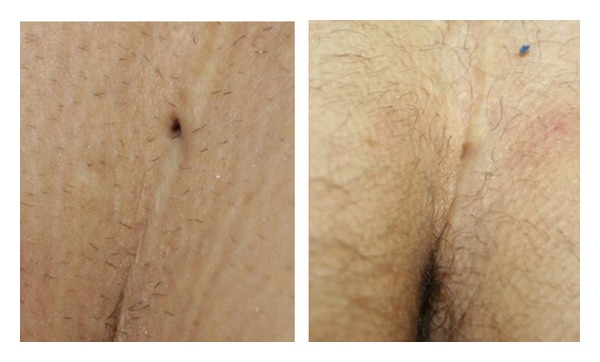
Pilonidal cyst: a complete resolution was achieved by the third session.

*Pilonidal Cyst*. A complete resolution was achieved by the third session (80 days after the first visit) in 3 patients treated. (Figure 4) After a follow-up period of 5 years, for the first patient treated, and one year, for the other two, no recurrence has been observed.

**Figure 5:**
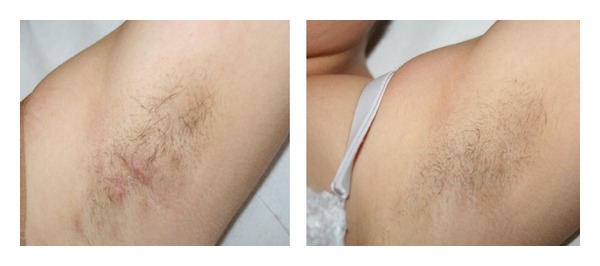
Hidradenitis suppurativa: complete resolution of pustular-papules progressive hair removal after 3 IPL treatments, bilaterally. Clinical stage I (Hurley's staging) and sartorius score of 24.

*Seborrheic Keratosis*. Superficial and small seborrheic keratoses responded well to IPL, whereas larger and/or deeper lesions may require a CO_2_ laser or other treatment. Dermoscopy is useful either to confirm diagnosis or to demonstrate a change in lesion colour from brown to grey immediately after treatment, thus predicting a good response to the treatment. Seborrheic keratosis was usually resolved with a mild inflammation and a complete recovery within 30 days after an average of two treatments (Figure 5).

**Figure 6:**
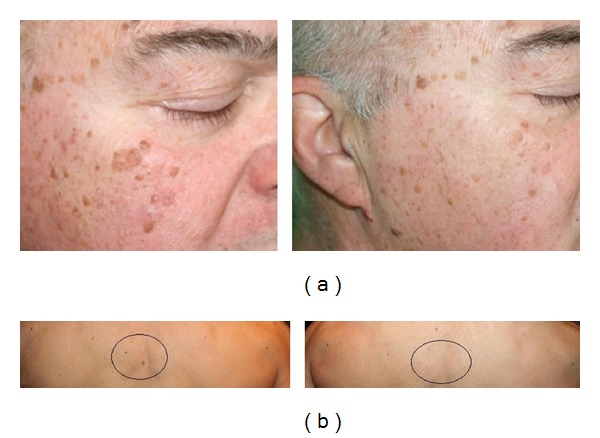
Seborrheic keratosis: (a) significant reduction of multiple seborrheic keratoses of the face after 2 IPL sessions, (b) seborrheic keratoses of the back disappeared after 2 IPL sessions.

**Figure 7:**
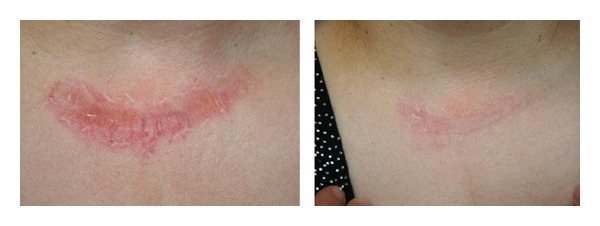
Hypertrophic scar: significant reduction of vascular component in the thicker areas before and after 3 IPL treatments.

*Hypertrophic Scars and Keloids*. Dermoscopic images revealed a significant reduction of vascular component in the thicker areas. Scars flattened and became smaller after three sessions. (Figures 6 and 7) All in all, good results were achieved, even though lengthy treatment (several months) is needed. The obtained results were stable during the follow-up. In one out of 5 cases of keloids, the lesion has resumed its growth phase.

**Figure 8:**
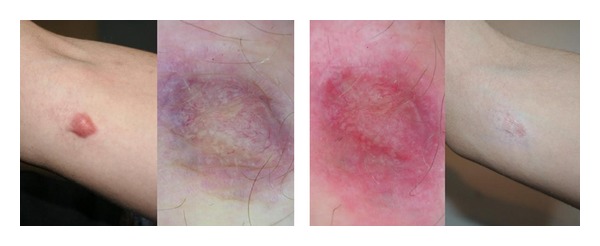
Keloid: scar after three sessions of IPL. Dermoscopy performed immediately after the first treatment showed a variation of the color from red-blue to red.

*Becker's Nevus*. A progressive hair removal and a reduction of the hyperpigmented area were achieved to the good satisfaction of both patients (Figure 8).

**Figure 9:**
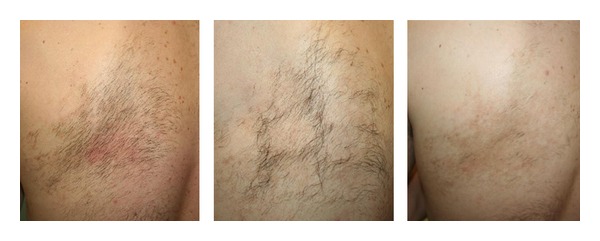
Becker's nevus: a progressive hair removal and a reduction of the hyperpigmented area were achieved to the good satisfaction of the patient.

*Hidradenitis Suppurativa*. At the end of the suggested scheme protocol, hidradenitis suppurativa was completely removed in both its inflammatory and painful components; hair removal was also achieved (Figure 9).

**Figure 10:**
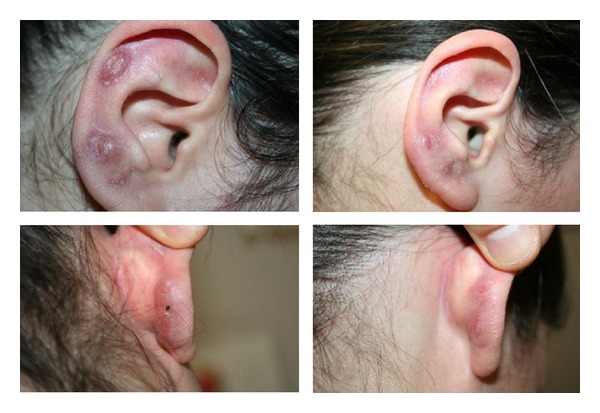
Sarcoidosis: significant reduction of the nodules with diminution of the painful sensation after 3 IPL treatments.

*Sarcoidosis*. A significant reduction of the vascular component and in the consistency of the lesions was achieved, thus leading to pain disappearance (Figure 10).

## 4. Discussion

In this study, we report on our good results achieved with almost all 58 patients affected by different dermatological conditions. With the aim of providing more exhaustive details, we will briefly discuss each condition separately.

*Rosacea*. Treatment of clinical manifestation of rosacea usually involves lasers such as argon, pulsed dye, Nd:YAG, CO_2_, and KTP, frequently causing burns, pain, and outcomes such as scars and significant hyperpigmentation due to incautious assessment of the lasers' photophysical parameters.

The ability to choose the duration of pulses makes IPL a versatile tool in the treatment of rosacea. The possibility of different filter settings (515, 550, 560, 570, and 590 nm) allows a wider selection of the range colour of the vascular system. A surface of 2.8 cm^2^ can be treated with a single shot, in contrast to the pulse dye laser (1.96 cm^2^ or 0.78 cm^2^) and argon (3 mm^2^). The larger surface offers greater efficiency, in terms of reducing treatment sessions, and less discomfort for the patient. Because it is able to divide the energy into two or three pulses with different delays between one pulse and the next, IPL allows the skin to cool down with minimal side effects [5].

Since the treatment is relatively unpainful, it can be carried out in the absence of anaesthesia. Immediate response usually presents as a slight erythema and a purple colouring which spontaneously resolves within 24–96 hours [5].

In a pilot study conducted by Mark et al., a 30% reduction of blood flow, a 29% reduction of telangiectasias, and a 21% reduction of erythema have been observed after five sessions of IPL. Taub et al. noticed a reduction of 83% of erythema, a reduction of 75% of flushing, and an improved skin texture [5, 6]. A 2008 study performed by Papageorgiou et al. noted the effectiveness of IPL in the treatment of rosacea disease of phase I. It showed a significant improvement of erythema, telangiectasias, and flushing. Severity was reduced and persistent results at 6 months with minimal side effects were obtained [2]. Reduction in the mechanical integrity of connective tissue of the dermis surface, responsible for passive dilatation of the blood vessels and thus resulting in erythema, telangiectasia, release of inflammatory mediators, and the formation of inflammatory papules and pustules, seems to play a key role in the treatment of rosacea. Moreover, IPL can improve rosacea through the ablation of its abnormal vessels and through the collagen remodelling of the dermis.

Furthermore, IPL determines a significant reduction of inflammation and in the number of active sebaceous glands, thus blocking, with great effectiveness, the altered process of keratinization [6].

In our study, all patients achieved significant results with 2 to 5 sessions of treatment.

*Port-Wine Stain*. Lasers such as the pulse dye laser, Nd:YAG, alexandrite, and the diode laser are the most used ones in the treatment of PWS [7, 8].

Currently, the first-choice treatment for PWS is represented by the pulsed dye laser; unfortunately, it cannot completely remove PWS. The energy emitted reaches only superficial vessels, thus resulting in a decreased amount of available light to hit the deeper ones (shadow effect). Because of this effect, hyper- and hypopigmentation and atrophic and hypertrophic scars may result after treatment [10]. When a PWS, especially with nodular component, is treated with an external light source, the main goal is to reach the vessels localized at the lower surface. IPL, thanks to its variability of pulse and fluence and to its possibility to divide the energy into different pulses, allows an additional heating which leads to coagulation of blood vessels of different diameter and different depth [1, 8]. Raulin et al. reported a 70–99% resolution of pink-coloured PWS after 2.9 treatments, of red PWS after about 1.4 treatments, and of purple PWS after an average of 2.4 sessions. In a study by Ozdemir et al., 37 patients with PWS were evaluated with results of up to 100% in 7 patients and 70–99% in 14 patients. In fact, IPL can be considered an effective treatment option. However, IPL systems require considerable experience and should be conducted with the aid of a good dermoscopy in order to determine the type of vessels to treat [34].

*Disseminated Porokeratosis*. Potential therapies include topical 5-fluorouracil, oral retinoids, CO_2_ laser, pulse dye laser, Nd:YAG, cryotherapy, dermabrasion, surgical excision, and imiquimod, or a combination of several therapies simultaneously [11–14].

In cases of superficial actinic porokeratosis, IPL proves to be a valid therapeutic option by determining a destruction of the pigment without risk of scarring or other side effects.

*Pilonidal Cysts and Hidradenitis Suppurativa*. According to the literature, laser technology applied in such cases includes CO_2_ laser and Nd:YAG. For its photocoagulative action, CO_2_ laser treatment produces a precise wound with minimal blood loss, leaving a surgical field clean and dry, but it is able to coagulate large vessels and requires a long recovery period [16–19].

IPL may represent a valid option for such lesions. The broad light spectrum is absorbed by the hair shaft, generates heat, and destroys the hair follicle. IPL acts on the melanin of the hair follicle causing necrosis of the follicle within the cyst. Similarly, it acts on the hairs of the surrounding area in order to reduce recurrence. Moreover, IPL has proven to be a powerful anti-inflammatory treatment able to eliminate the chronic inflammation within the cyst [19]. In 2011, Highton et al. selected 18 patients affected by HS and treated one axilla, groin, or inframammary area with intense pulsed light two times per week for 4 weeks using a harmony laser, whereas the contralateral side received no treatment and was used as a control. A significant improvement in the mean examination and its persistence at 12 months led patients to report high levels of satisfaction. No concurrent improvement on the untreated control side has been observed. This small study suggests that intense pulsed light may be an effective treatment for HS. Although only a few data have been reported so far, results suggest efficacy and safety and the absence of side effects [26].

*Seborrheic Keratosis*. In previous studies, lasers have been demonstrated to be effective in the treatment of seborrheic keratosis, such as alexandrite (755 nm) and diode laser [21].

No studies on the use of pulsed light for the treatment of seborrheic keratosis have been published so far. Thanks to its broad spectrum of action, it is possible to select the specific wavelength to act selectively on the melanin pigment of seborrheic keratosis. Immediately after the treatment, a change in colour from brown to grey is observed at dermoscopic evaluation and this represents a sign of success of the performed procedure [34]. Subsequently, keratosis tends to disappear completely without residual erythema. The treatment is, however, limited to superficial and small seborrheic keratoses.

*Hypertrophic Scars and Keloids*. The pulse dye laser has been reported to produce long-term improvements in the appearance of hypertrophic scars. A very recent pilot study has demonstrated the effectiveness of IPL in wound healing after suture removal. The basic mechanism is not yet fully understood but most probably an action on vascular proliferation, essential for the growth of collagen, and on pigmentation resulting from scar formation is involved [22]. Despite the wide use of IPL in various skin diseases, only a few studies demonstrating its effectiveness on hypertrophic scars have been published to date. Wavelengths around 1200 nm are absorbed by the water within the dermis thus triggering a reaction that leads to cytokine stimulation of collagen fibres of types I and III and elastin. The absorption peak of the collagen fibres is found to be from 400 nm to 600 nm. The heating of the collagen fibres by the IPL leads to their contraction, with a clinically detectable improvement in the texture. The IPL, in contrast to other treatments, is not invasive and has very few side effects. Bellew et al. have shown that the IPL is as effective as the long pulse dye laser (595 nm), resulting in a greater softness of the scar. Kontoe et al. reported an improvement of more than 75% in the pigmentation of hypertrophic scars, 50% higher than that in the scars from asphalt, and 50% reduction in the size and thickness of hypertrophic scars. This is probably due to the inhibition of the action of the vessel caused by IPL on scar tissue and on the subsequent proliferation of collagen [22].

*Becker's Nevus*. Trelles et al. compared the effectiveness of the Erbium:YAG laser with the Nd:YAG laser in 22 Becker's nevus patients, 11 for each group. Up to now, there have been no studies on the treatment of Becker's nevus with IPL. Such treatment is able to produce synchronized single or multiple pulses with the possibility of varying the pulse duration. We can then select the appropriate wavelength, taking into account the main absorption spectrum of the pigmented structures (between 400 nm and 600 nm) and the right pulse duration to act efficiently on the hair follicle. We can operate on both components with excellent results. [23, 24].

*Sarcoidosis*. In his systematic review on the use of pulsed dye laser in the treatment of inflammatory skin diseases published in 2013, Erceg A reported on five case reports of PDL treatment for cutaneous sarcoidosis/lupus pernio [28–33]. In our experience, IPL has been proven to have a significant effect on the vascular component of granulomata. Even though IPL could not definitely treat cutaneous sarcoidosis, a great improvement of patients' pain and symptoms could be achieved.

## 5. Conclusions

According to the literature, the effectiveness of IPL has now been well demonstrated. Its versatility, in contrast with many single-laser spectrums, has led to its rapid spread in different clinical scenarios, while the wide range of wavelengths allows us to use these devices for a broader range of clinical conditions. However, we would like to underline how the use and effectiveness of the IPL are strongly related to the operator's experience. Apart from facilitating excellent outcome, the broad spectrum of wavelengths used and the high number of parameters can affect the final result and increase the risk of side effects. The daily use of these devices will surely increase clinical experience and provide new information, thus enhancing long-term results and improving IPL effectiveness.

## Figures and Tables

**Table 1:** Clinical data.

	Number of patients	Gender	Mean age (range)
Rosacea	10	5 M, 5 F	51.6 (38–62)
Port wine stain	10	7 M, 3 F	52.1 (8–52)
Disseminated Porokeratosis	10	2 M, 8 F	58.3 (41–70)
Pilonidal cyst	3	3 M	24.6 (18–34)
Seborrheic keratosis	10	6 M, 4 F	61.7 (35–83)
Hypertrophic scar/keloids	5/5	3 M, 2 F/2 M, 3 F	30.2 (21–37)/34.8 (27–43)
Becker's nevus	2	2 M	29 (26–32)
Hidradenitis suppurativa	2	1 M, 1 F	32 (26–38)
Sarcoidosis	1	1 F	26
Total	**58**	**32M, 26F**	**42.7 (8–83)**

**Table 2:** IPL setting for each off-label dermatological disease treated.

	Filters	Number of pulses	Pulse duration	Delay	Fluence	Number of sessions
Rosacea erythematotelangiectatic component	500	2	5–10 msec	10 msec	12–16 J/cm^2^	Up to 4
Rosacea papulopustular component	550	2	5–10 msec	10 msec	10–12 J/cm^2^	Up to 5
Port wine stain	500	2	5–10 msec	10 msec	13–16 J/cm^2^	Up to 5
Disseminated porokeratosis	550	2	5–10 msec	10 msec	10–12 J/cm^2^	Up to 4
Pilonidal cyst	550	3	5 msec	20 msec	7–9 J/cm^2^	Up to 3
Seborrheic keratosis	550	2	5–10 msec	10 msec	10–12 J/cm^2^	2
Hypertrophic scar and keloid pigmented component	550	2	5–10 msec	10 msec	10–12 J/cm^2^	Up to 5
Hypertrophic scar and keloid vascular component	500	2	5–10 msec	10 msec	14–17 J/cm^2^	Up to 6
Becker's nevus hair removal	550	2-3	5 msec	10–20 msec	7–9 J/cm^2^	Up to 4
Becker's nevus pigmented component	550	2	5–10 msec	10 msec	9–12 J/cm^2^	Up to 5
Hidradenitis suppurativa hair removal	550	2-3	5 msec	10–20 msec	7–9 J/cm^2^	Up to 4
Hidradenitis suppurativa inflammatory component	550	2	5–10 msec	10 msec	8–10 J/cm^2^	Up to 5
Sarcoidosis	500	2	5–10 msec	10 msec	12–16 J/cm^2^	Up to 4
